# Identification of long non-coding RNA in the horse transcriptome

**DOI:** 10.1186/s12864-017-3884-2

**Published:** 2017-07-04

**Authors:** E. Y. Scott, T. Mansour, R. R. Bellone, C. T. Brown, M. J. Mienaltowski, M. C. Penedo, P. J. Ross, S. J. Valberg, J. D. Murray, C. J. Finno

**Affiliations:** 1 Department of Animal Science, University of California, Davis, USA; 2 Department of Population Health and Reproduction, University of California, Davis, USA; 3 Department of Clinical Pathology, College of Medicine, Mansoura University, Mansoura, Egypt; 4 Veterinary Genetics Laboratory, University of California, Davis, USA; 5 Large Animal Clinical Sciences, Michigan State University, College of Veterinary Medicine, East Lansing, USA

**Keywords:** Long non-coding RNA, Equine transcriptome, Intergenic

## Abstract

**Background:**

Efforts to resolve the transcribed sequences in the equine genome have focused on protein-coding RNA. The transcription of the intergenic regions, although detected via total RNA sequencing (RNA-seq), has yet to be characterized in the horse. The most recent equine transcriptome based on RNA-seq from several tissues was a prime opportunity to obtain a concurrent long non-coding RNA (lncRNA) database.

**Results:**

This lncRNA database has a breadth of eight tissues and a depth of over 20 million reads for select tissues, providing the deepest and most expansive equine lncRNA database. Utilizing the intergenic reads and three categories of novel genes from a previously published equine transcriptome pipeline, we better describe these groups by annotating the lncRNA candidates. These lncRNA candidates were filtered using an approach adapted from human lncRNA annotation, which removes transcripts based on size, expression, protein-coding capability and distance to the start or stop of annotated protein-coding transcripts.

**Conclusion:**

Our equine lncRNA database has 20,800 transcripts that demonstrate characteristics unique to lncRNA including low expression, low exon diversity and low levels of sequence conservation. These candidate lncRNA will serve as a baseline lncRNA annotation and begin to describe the RNA-seq reads assigned to the intergenic space in the horse.

**Electronic supplementary material:**

The online version of this article (doi:10.1186/s12864-017-3884-2) contains supplementary material, which is available to authorized users.

## Background

Long non-coding RNA (lncRNA) are transcripts usually defined as larger than 200 nt and lacking a productive open reading frame (ORF) for translation. These transcripts typically function in regulation of mRNA expression levels [1], nuclear organization [2] and various developmental processes including differentiation [3]. LncRNA are often found in low abundance compared to protein-coding genes [4] and exhibit shorter transcript sizes and less exon diversity [5]. Due to their low sequence conservation across species [6], their tissue-specific nature within species [7], and a lack of knowledge regarding their function, lncRNA are difficult to identify and validate. They have been shown to exhibit more variability in expression than protein-coding genes [8] and the number of lncRNA detected is affected and increases when more individuals are used to formulate the lncRNA database [9]. Thus, having transcript expression profiles from several tissues collected from multiple individuals is paramount in detecting the maximum number of lncRNA.

The transcriptomic landscape of the horse is mainly defined by RNA sequencing (RNA-seq). Recently, an equine transcriptome, defined by RNA-seq datasets covering eight tissues, from 59 individuals was published [10]. However, the filtering processes focused on protein-coding transcripts from these RNA-seq datasets and resulted in a discard of 16% of transcription due to lack of support by any gene models. Another 20% of the transcription was directed towards novel transcripts with undetermined annotation. In an effort to further characterize this uncertainty, genetic features other than protein-coding transcripts should be annotated. In the horse, there is a lack of annotation for functional elements beyond protein-coding transcripts and conservation of lncRNA in other species cannot be relied upon for this annotation. Therefore an equine specific lncRNA database is required. In this study, we annotate lncRNA transcripts and thereby increase the proportion of transcriptome that is annotated in the horse.

Previous work attempting to capture the breadth of lncRNA within the horse is limited to one recent publication identifying several potential lncRNA in peripheral blood mononuclear cells [11] using polyA-captured RNA-seq libraries. Most efforts towards identifying noncoding RNAs has gone to miRNA identification [12–14]. There are, however, over 4,000 lncRNA transcripts predicted by ENSEMBL and NCBI represented in our transcriptome that we have considered as input for our annotation pipeline. Our pipeline for annotating candidate lncRNA integrates eight tissues: the cerebellum, brainstem, spinal cord, retina, skeletal muscle, skin, and the embryonic inner cell mass (ICM) and trophectoderm (TE). Among these tissues, there is a mixture of rRNA depleted and polyA-captured RNA-seq library preparations, strand-specific libraries and a range of library depths from over 200 million reads to under 20 million reads per tissue. This pipeline serves as an additional tool to the equine protein-coding transcriptome annotation pipeline and maximizes the utility of the RNA-seq datasets.

## Methods

### Input categories of reads

The initial inputs into this lncRNA pipeline were direct products of the transcriptome annotation pipeline based on RNA-seq from eight equine tissues: brainstem, cerebellum, spinal cord, retina, skeletal muscle, skin and embryo ICM and TE, originating from 59 horses [10]. There were five categories of input, four originating from the initial equine transcriptome pipeline and considered novel or intergenic, and the fifth coinciding with lncRNA already predicted by NCBI and ENSEMBL. A full description of these categories can be found in the equine transcriptome paper [10]. Briefly, transcript categories novel I, II, and III inputs were considered novel transcripts with decreasing levels of supportive evidence, ranging from support from other equine annotations (novel I) to support from orthologous gene models or gene prediction models (novel II) and finally lacking any support but having a conserved ORF (novel III). The novel transcript categories were previously filtered according to several criteria supporting the likelihood that these transcripts were from protein-coding genes, including the presence of an ORF, length exceeding 200 bp, not being completely contained within introns of annotated genes and transcript not representing isoforms of a gene that were under-supported by RNA-seq evidence. The intergenic category of transcripts represented transcripts that lack any supportive evidence or ORFs. The final input group included 3956 transcripts from our refined transcriptome that have exonic overlap with previously predicted lncRNA (known lncRNA) from NCBI and ENSEMBL of which 2634 were in the novel I, 117 in the novel II and 136 in the novel III input. The total number of transcripts in all five groups before filtering was 62,216 (Table 1).

**Table 1:** General lncRNA statistics and the number of candidate lncRNA transcripts that passed through each filter. Filter numbers correspond to Fig. 1

		Novel I	Novel II	Novel III	Intergenic	Known lncRNA	total
Initial number of transcripts		8459	7494	6687	38,507	3956	62,216
Number of lncRNA	F1	7193	3873	1128	15,686	3523	30,998
	F2	7193	3873	1128	15,281	3523	28,503
	F3	4408	3102	726	15,162	2593	24,029
	F4	3334	2475	639	13,804	2011	20,800
Average Length (kb)		3.2	3.2	2.3	1.2	3.8	-
Average TPM		18.3	28.2	3.9	1.8	4.0	-
GC%		45.3	45.1	48.7	43.1	44.4	-
Total bp		10,604,817	7,870,739	1,465,708	16,880,112	5,658,390	-

### Step-wise filtering of reads

**Fig. 1:**
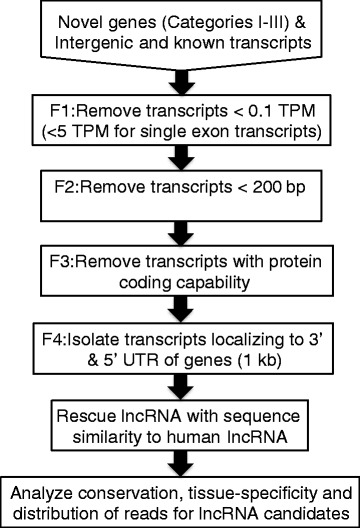
Filtering pipeline used for candidate lncRNA. The inputs correspond to products of the protein-coding transcriptome [10]

Transcripts from novel I, II, III, intergenic, and known lncRNA categories underwent step-wise filtering using four filtration steps (Fig. 1).

#### Filter 1: removal of lowly expressed transcripts

All transcripts were initially filtered based upon on a mean expression threshold across all tissues of 0.1 transcripts per million (TPM), as calculated by Salmon [15] after backmapping each tissue RNA-seq library to the candidate lncRNA transcripts. Additionally, more stringent expression thresholds of 5 TPM in any given tissue were applied to single exon transcripts. Similar thresholds were used in a recent human lncRNA annotation [16].

#### Filter 2: removal of short transcripts

Any transcript less than 200 bp was removed, however this only applied to the intergenic category of transcripts, as the novel I, II and III inputs were already filtered for size [10].

#### Filter 3: removing transcripts with protein-coding capability

Protein-coding capability was assessed using HMMER [17] and BLASTP [18] on the ORF sequences predicted by Transdecoder [19]. Transdecoder and HMMER software were used with the default parameters. Transcripts with an ORF of at least 100 amino acids and any predicted protein motif or BLASTP hit with *p*-values less than 10^−3^ were removed [16, 20]. An ORF length of 100 amino acids was used because lengths below 100 amino acids severely increase the number of false positives [19]. The reference protein databases used for HMMER and BLASTP were the Pfam-A [21] and Uniprot databases [22], respectively.

#### Filter 4: isolating and removing any transcripts within 1 kb of an annotated gene

Transcripts falling within 1 kb up- or down-stream of any likely protein-coding gene and on the same strand in the “refined transcriptome” provided by Mansour et al. [10] were isolated and removed. This was a filter adapted from the human lncRNA pipeline [16] and was particularly applicable in the horse due to the frequent incomplete UTR annotation of protein-coding genes resulting in gene fragments flanking genes on the same strand. The transcriptome used here was the published “refined transcriptome” with the candidate lncRNA post-filter 3 removed. This was performed using the bedtools intersect program [23] and by extending genomic start and stop coordinates by 1 kb.

### Rescue of filtered lncRNA

Because of an observed loss during the protein-coding capability filter of orthologous lncRNA that were well annotated in human, all transcripts removed by filter 3 had BLASTN performed against human lncRNA to rescue the well documented candidate lncRNA. A *p*-value of 10^−5^ was used and any transcripts with over 25% query coverage and 75% identity were retained.

### Conservation of lncRNA

Conservation of the equine lncRNA sequences relative to human lncRNA compared to their protein-coding counterparts were analyzed using BLASTN. The equine candidate lncRNA sequences and protein-coding transcripts were blasted against a concatenated file of human lncRNA and protein-coding transcripts (from ENSEMBL), termed human transcriptional products. A BLASTN measure of conservation was generated by multiplying the percent identity and percent coverage for each hit and calculating the cumulative frequency of transcripts attaining each measure of this conservation. Similar procedures were also conducted with mouse, cow and pig transcriptional products.

### Tissue specific expression of lncRNA

Tissue-specific expression of lncRNA was assessed by comparing the cumulative TPM of tissue-expressed transcripts, hierarchical clustering of lncRNA expression in tissues, and identification of unique expression. Tissue-expressed transcripts were selected as transcripts with a TPM greater than 0.1. The cumulative TPM of tissue-expressed lncRNA was compared to that of expressed protein-coding transcripts in the same tissue. The comparison was presented as a scatter diagram of pie charts in relation to the numbers of expressed lncRNA and protein-coding transcripts using the pies function of “caroline” R package [24]. For hierarchical clustering, a subset of lncRNA (1450 transcripts), with a sum and standard deviation of TPM across all tissues above 100 and 50, respectively, were selected. Bi-clustering was performed by Pearson correlation for expression of selected transcripts and Spearman correlation of expression profiles in tested tissues using the heatmap.2 function of “gplots” R package [25]. Finally, specific presence of a lncRNA transcript was defined by an expression of at least 0.1 TPM in one tissue, with less than 0.1 TPM in all other tissues. Specific absence of a lncRNA transcript was defined by an expression of less than 0.1 TPM in one tissue with TPM values of above 0.1 in all other tissues. Results were graphically presented using “ggplot2” R package [26].

## Results

### Filtering of lncRNA

**Fig. 2:**
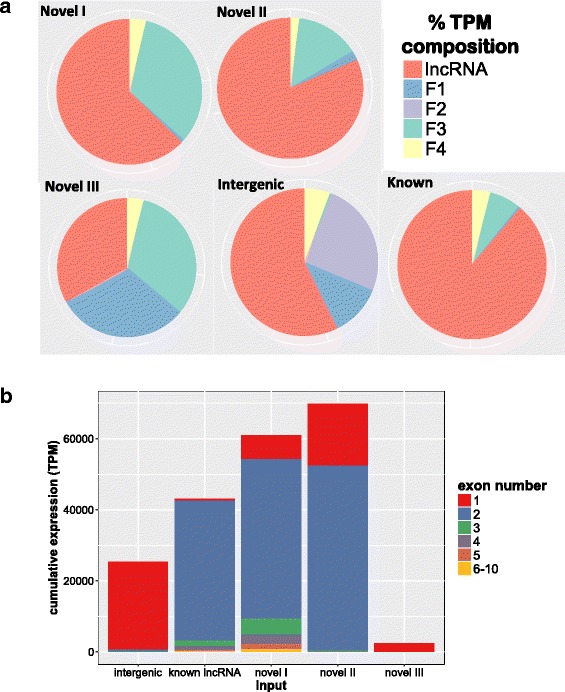
Different behavior seen by inputs novel I, novel II, novel III, intergenic and known lncRNA transcripts during and post filtering. **a** The amount of transcriptional output removed by each filter (F1, F2, F3 and F4, as labeled in Fig. 1), where the whole pie represents all the transcriptional output of that input and each wedge represents the cumulative TPM removed by each filter. **b** The exon diversity relative to the total cumulative TPM provided by each input post-filtering

Overall, 62,216 transcripts were used as input and, after applying our pipeline for lncRNA discovery, we identified 20,800 candidate lncRNA. Removal of lowly expressed transcripts (filter 1) imposed the greatest exclusion of transcripts from the novel III input, where 83% of the initial transcripts from novel III were removed (Table 1, Fig. 2a). Removal of transcripts shorter than 200 nt, filter 2, only eliminated 3% of the intergenic transcripts post filter 1, but removed around a quarter of the initial transcriptional output. Removal of protein-coding transcripts (filter 3) had the largest impact on novel I and III inputs, with 39% and 36% of the transcripts following filter 2 excluded, respectively. Removal of the likely fragmented UTRs (filter 4), had a large impact on the novel I transcript count, where it removed 24% of the novel transcripts post filter 3. Filter 4 eliminated progressively less transcripts in the inputs novel II, novel III and intergenic, with 21, 12, and 9% of the transcripts post filter 3 being excluded, respectively, along with little removal of transcriptional output (Table 1, Fig. 2a). The previously identified lncRNA were most impacted by filter 3, where 26% of the post filter 2 transcripts were removed, and by filter 4, where 22% of post filter 3 transcripts were removed. The final step for rescuing the well-annotated human lncRNA resulted in a return of 134 transcripts (3% of the transcripts removed by filter 3) to the lncRNA database. Most of the 20,800 candidate lncRNA identified came from the intergenic input dataset (Table 1), with most expression of candidate lncRNA coming from the novel II input (Fig. 2b).

### Conservation of lncRNA

**Fig. 3:**
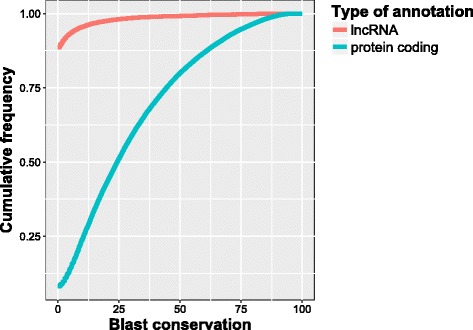
Sequence conservation of equine lncRNA and protein-coding transcripts relative to human transcriptional products. Blast conservation represents the BLASTN identity multiplied by the BLASTN coverage of a given transcript. The cumulative frequency represents the percentage of lncRNA transcripts obtaining a BLASTN conservation measure equal to or less than the indicated x-axis value

Relative to human transcriptional products [27], the equine lncRNA demonstrate no sequence conservation compared to their protein-coding counterparts. The cumulative frequency referred to in Fig. 3 represents the percentage of BLASTN hits attaining or having less than the BLASTN conservation measure on the x-axis. For instance, as is demonstrated by the elevated starting position of the lncRNA conservation line, 88% of candidate lncRNA transcripts had no significant BLASTN hit compare to the 8% of protein-coding not receiving a BLASTN hit. Additionally, a cumulative 90% of these candidate lncRNA attained a 40 times lower BLASTN conservation compared to the protein coding transcripts (Fig. 3). Similar results were seen with the mouse, cow and pig transcriptional products (Additional file 1). While sequence conservation appears to be low, there does appear to be positional conservation of lncRNAs. Specifically we noted five well-characterized lncRNA demonstrating this conservation in Table 2, despite having BLASTN sequence identities below 80%. Further examples can be found in Additional file 2.

**Table 2:** Five examples of equine lncRNA compared to human lncRNA in terms of relative position to surrounding genes and BLASTN percent identity and percent coverage of the equine lncRNA relative to the human counterparts

Proposed lncRNA	Horse coordinates	Distance to nearest gene in horse	Human coordinates	Distance to nearest gene in human	% identity	% coverage
*Gas5*	chr5:9,536,77 0–9,543,475	390 (5’antisense *ZBTB37*)	chr1: 173,863,900–173,867,989	93 (5’antisense *ZBTB37*)	70	43
*NEAT1*	Chr12:255851 09–25613745	7235(3′ *FRMD8*)	Chr11:65,422,798–65,445,538	9274 (3′ *FRMD8*)	74	63
*LINC00884*	Chr19:31292750–31300597	1030 (5′ antisense to *ATP13A3*)	chr3:194,487,140–194,488,545	Overlap with *ATP13A3* (antisense)	68	16
*TSIX*	chrX: 55,214,315–55,2 43,223	Complete overlap (antisense) to *XIS7* lncRNA	chrX:73,792,205–73,829,231	Overlap with *XIST* lncRNA (antisense)	75	54
*EPHA5-AS*	chr3:68,892,305–68,911,651	131 (5′ antisense EPHAS)	chr4:65,669,961–65,693,386	382 (5′ antisense *EPHA5*)	77	91

### Tissue and library patterns of the candidate lncRNA

**Fig. 4:**
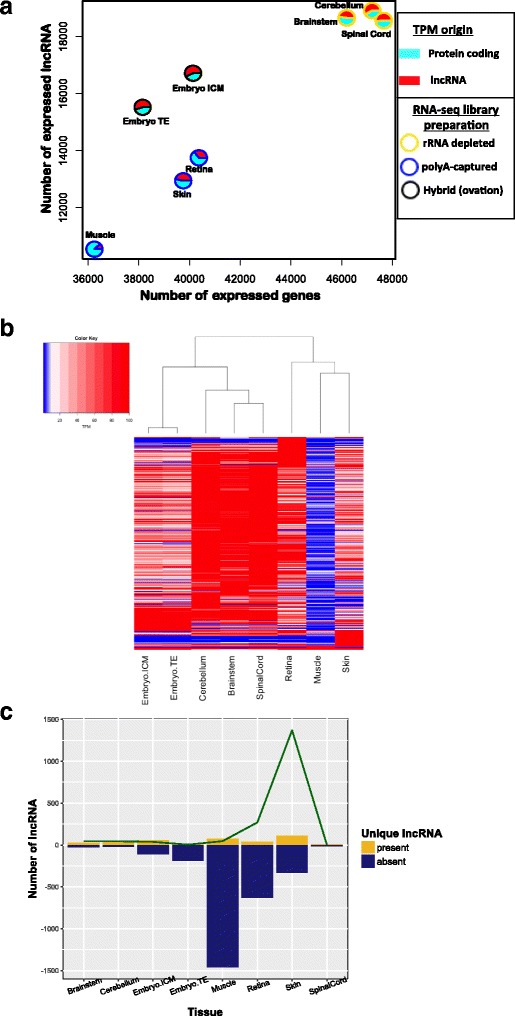
Tissue and RNA-seq library preparation effects on lncRNA detection and expression. **a** There is a positive relationship between the number of annotated genes and candidate lncRNA detected in each tissue; the pie charts represent the cumulative TPM of that tissue with the turquoise correlated to the expression of the protein-coding transcripts and red to the candidate lncRNA expression. The pies outlined in *yellow* were rRNA-depleted RNA-seq libraries, pies outlined in *black* were Ovation RNA-seq libraries and the pies outlined in *blue* were the polyA-captured RNA-seq libraries. **b** The hierarchically clustered heatmap also shows clustering on a tissue and RNA-seq library level. **c** There is a distinguishable difference in the number on lncRNA that seem to be unique to a given tissue, with the skin having the largest number of unique lncRNA and the highest cumulative expression associated with its unique lncRNA. The green line represents the cumulative TPM of all the uniquely present lncRNA, divided by 5 for scaling

Due to the inherent tissue-specific nature of lncRNA, we expected to observe patterns correlating to tissue type along with potential effects of the library preparation methods utilized. Briefly, the spinal cord, brainstem and cerebellum samples were rRNA depleted, the muscle, retina and skin libraries were polyA-captured and the embryonic tissues were a variation of the two, using Ovation RNA-seq System V2 (NuGEN, San Carlos, CA, USA) [10]. Due to this variety of library preparations across tissue types, discriminating between the contributions from the library preparation or the tissue type on the lncRNA patterns observed cannot be accurately determined. However, the polyA-captured RNA-seq library preparations do seem to demonstrate less detection of candidate lncRNA on several levels including total number (Fig. 4a), expression (Fig. 4b) and number of solely absent candidate lncRNA (Fig. 4c) relative to the rRNA depleted RNA-seq libraries.

Additional to the obvious tissue and library preparation effects on expression of lncRNA candidates, there were also effects on diversity of lncRNA detected per tissue. A positive relationship between the expressed protein-coding transcripts and candidate lncRNA was found. The three rRNA depleted libraries and tissues - spinal cord, brainstem, and cerebellum - demonstrated the largest number of coding and non-coding transcripts. On the other hand, the retina, skin and muscle, three poly-A libraries, displayed the least number of both. The ratio of lncRNA to protein-coding transcripts was highest in embryonic TE (0.5) and lowest in the muscle (0.26) (Fig. 4a). Based on expression patterns of the more robust and variable candidate lncRNA, we observed clustering of the tissues similar to clustering seen with protein-coding transcripts [10]; however, again these tissues were clustering in a manner that is dependent upon their library preparation (Fig. 4b). Although the skin demonstrated relatively low numbers of candidate lncRNA detected, it had the most candidate lncRNA showing tissue specificity, with 110 candidate lncRNA of the 13,750 detected (0.8%) considered as uniquely present in the skin (Fig. 4c). Additionally, the skin, had a subset of uniquely present transcripts, which exhibited the highest cumulative TPM of all these unique transcripts, with a cumulative total of 6851 TPM. Tissue-specific expression values for all lncRNA and protein-coding transcripts used can be found in Additional file 3, with the browser extensible data (BED) and gene transfer format (GTF) tables for the lncRNA in Additional files 4 and 5, respectively.

## Discussion

In this study, we relied on known conventions of lncRNA, including expression and transcript length, to extract the most likely lncRNA candidates from the RNA-seq datasets. As expected, we obtained a subset of transcripts showing lower expression, less exon diversity and shorter transcript lengths than protein-coding transcripts, as observed in lncRNA databases from several other species [4, 5, 28]. Species with less well-defined transcriptomes like the cow and dog, have 9778 [29] and 12,370 [16] annotated lncRNA transcripts, respectively. Species with better defined transcriptomes, such as the human, rhesus and mouse, have more - 31,738, 21,908 and 34,643 annotated lncRNA transcripts [16], respectively. These studies used datasets range from 1 individual (cow) to 27 individuals (human) and 10 tissues (dog) to 27 tissues (human). In this dataset, the final number of annotated lncRNA transcripts in the horse was 20,800 across 59 individuals and 8 tissues.

We analyzed the behavior of the five inputs, novel I, II, III, intergenic and previously recognized lncRNA, separately through the filtration process to assess whether the filters removed the expected number of transcripts, given the previously known composition of the five inputs. The novel I, novel II and novel III categories of transcripts had decreasing levels of expression, exon diversity, and supportive evidence from other equine databases or RefSeq gene models [10], thus we expected novel I transcripts to largely represent protein-coding transcripts, while novel II and novel III transcripts would contain more lncRNA candidates. The largest contribution of candidate lncRNA was expected to come from the intergenic input due to its 1.7 fold higher initial transcript input over the combined novel I, II, and III inputs and due to lack of gene-model support [10]. Despite the intergenic input having over 17-fold more candidate lncRNA than the novel II input, the novel II input had the highest cumulative TPM of lncRNA (Fig. 2b) and mean expression of the candidate lncRNA (Table 1). The supportive evidence that defined the novel II input is comprised of RefSeq gene models, of which lncRNA models are also included [30], thus novel II input may represent high expressing lncRNA already annotated in other species. The novel I input was expected to contain the most protein-coding transcripts, and it did have the largest removal of transcripts from filter 3 (Table 1, Fig. 2a). However, the novel I input had the most overlap with the previously recognized equine lncRNA, as evidenced by the similar exon composition between the known and novel inputs, and thus explaining the novel input expression contribution to this lncRNA annotation (Fig. 2b). The novel III input provided the least number and lowest expression of candidate lncRNA; this was due to the strict filtering on single exon transcripts (filter 1) and the low expression of candidate lncRNA. Regarding the previously identified equine lncRNA, only 50% remained after the filtering process, with much more of its transcriptional output retained (Table 1, Fig. 2b). The known lncRNA group was largely impacted by the protein-coding filter 3 and their proximity to protein-coding models (filter 4), all together suggesting that a large proportion of them could represent rare isoforms or gene fragments of protein-coding transcripts. Overall, the intergenic input showed the most even proportion of transcripts removed with each successive filter (except filter 2, which removed very little) with regards to initial transcriptional output (Fig. 2a). The behavior of different inputs through the filters, confirm the intended efficacy of each of the filters and further support the remaining candidate lncRNA.

Degree of conservation was another means of distingushing our group of candidate lncRNA from protein-coding transcripts. Compared to protein-coding transcripts, lncRNA demonstrate much less sequence conservation between species [31], with conservation being allocated across several other perspectives, such as regulation, position or secondary structure. As expected, we observed approximately 40 times less sequence conservation of a cumulative 90% of the hits between equine lncRNA and their protein-coding transcripts (against human transcriptional products), along with much more variable levels of conservation across the protein-coding transcripts (Fig. 3). Examples of positional conservation with relatively low sequence identity but similar position relative to surrounding genes include *Gas5, NEAT1, LINC00884, TSIX* and *EPHA5-AS* (Table 2), highlighting an alternative level of conservation that may be exhibited by the candidate lncRNA. Further avenues for identifying database-wide conservation on structural levels would be beneficial; however such large-scale software for RNA secondary structure or RNA interactions is not yet available.

Although there is an obvious combined effect of tissue-specificity and RNA-seq library preparation on lncRNA detection, a non-confounding study design is required to detect effects of both factors separately. When comparing the ratios of annotated lncRNA to protein-coding transcripts, the central nervous system (CNS), and thus the rRNA depleted RNA-seq libraries, group together with the largest amounts of lncRNA and protein-coding transcripts. Despite the similarities we would expect between the retina and CNS tissue [32], the retina seems to cluster more with the skin. This could be due to RNA-seq library type, and it may also be due to the low depth of reads and limited individuals composing both tissues’ RNA-seq libraries. The fewer lncRNA annotated in the muscle could relate to the more homogeneous population of cells sequenced compared to the other tissues or from the muscle having an inherently smaller transcriptome, as seen in other species [33]. Also technical issues such as PCR amplification or fragmentation can contribute to the bias [34]. Similar reasoning can also be applied to the skin and retina, as they are composed of single-end reads, which are known to show much more frequent instances of computationally detected read duplicates [34]. Given the candidate lncRNA to protein-coding ratios, the CNS and embryonic tissues exhibited larger than expected contributions of lncRNA expression to the total transcriptional output of the tissue. However the high proportion of lncRNA transcription in most of these tissues corroborate with others, emphasizing the functional impact of lncRNA in these tissues [4, 35–37]. The overall positive relationship seen between number of protein-coding transcripts and candidate lncRNA can also be seen in the lncRNA distribution across chromosomes, similar to the distribution of annotated genes in the recent equine transcriptome paper [10], where the number of transcripts annotated per chromosome appears to be related to the size of the chromosome (Additional file 6). The tissues expressing more genes tend to also express more candidate lncRNA, with the expression of the lncRNA often being higher than the ratio of lncRNA to protein-coding transcripts.

The hierarchically clustered heatmap (Fig. 4b) further resolved the cumulative TPM shown in Fig. 4a and clustered tissues based upon a subset of highly expressing and variable candidate lncRNA. The CNS tissues clustered together and shared a relatively large group of high expressing candidate lncRNAs, a majority of which have not been assigned to a specific equine chromosome (chrUn). The retina clustered close by the CNS tissues due to some overlap of highly expressed lncRNA candidates, likely because it too represents central nervous system tissue. The embryonic tissues clustered with one another, similar to the pattern observed with annotated genes in these tissues [10]. However the clusters of highly expressed lncRNA shared between the two types of embryonic tissue were far smaller than that seen within the CNS. This could be due to the smaller number of individual samples or the library preparation underlying the embryonic tissues. The small amount of lncRNA expression seen in the muscle is similar to what is seen in bovine skeletal muscle [29], but again, could be resulting from the polyA-capture RNA-seq library preparation. The skin demonstrates distinct clustering from the rest of the tissues in the heatmap, most likely because of the presence of a small number of lncRNA that had high expression and tissue specificity to the skin. The high cumulative expression seen in the skin could be partially attributed to four different lncRNA with TPM values over 100. Three of these lncRNA are located on ECA5 and showed sequence identity of over 70% with ncRNA from other species, however the query coverage was approximately 10%. One of these lncRNA had a TPM value of 772 and was also overlapping an equine gibberellin-regulated protein predicted gene (XM_014739772.1) on the antisense strand, which demonstrated tissue specificity and comparable TPM values of 612 in the skin. This particular lncRNA also showed 78% identity (with 95% coverage, e-value = 1e-85) to a predicted, but uncharacterized lncRNA in *Canis Lupis* (XR_294613.1). Due to poor functional annotation of lncRNA and the use of various RNA-seq library preparation types, it was difficult to assess tissue-specific trends in equine lncRNA. However, we were able to demonstrate that RNA-seq library preparation, combined with tissue effects, impact lncRNA expression, detection and abundance.

The heterogeneity of the RNA-seq libraries underlying the present equine lncRNA annotation and the lack of replicates for any given library preparation have prevented conclusions about strictly tissue-specific or library preparation-specific trends in lncRNA expression or annotation. Beyond the clear effects of library preparations on several clustering algorithms (Fig. 4), the length of the reads, whether they were single-end versus paired-end and whether they were shorter (81 bp) versus longer (150 bp), also had an effect. Some of the skin tissue RNA-seq libraries, for example, were composed of short (81 bp), single-end reads, which are not considered ideal for lncRNA annotation [38]. This resulted in discernable gene fragments with high expression that the protein-coding capability filter was incapable of removing due to the short ORFs produced. Thus there is an overestimation in lncRNA expression and detection in the skin. Each library type and tissue, would benefit from a specific lncRNA annotation pipeline tailored to the idiosyncrasies of each RNA-seq library preparation. However, the most suitable method for extracting more definitive results regarding tissue specificity would be to ensure that all tissues had the same library preparation as well as read characteristics. Additionally, filtering of lncRNA in this pipeline was conservative, therefore the rare, low-expressing lncRNA candidates or the candidates harboring protein-coding potential or lying adjacent (on the same strand) to a protein-coding transcript may have been removed.

## Conclusions

This research has assigned annotation to transcriptional output of unknown composition in the horse. Our candidate lncRNA provide sources for 16% of the overall transcriptional output, with much higher expression contributions in certain tissues. We expanded upon and further refined the previously annotated equine lncRNA, from 3965 transcripts to 20,800 transcripts. This subset of transcripts showed a profile similar to other documented lncRNA databases with transcripts exhibiting low expression, low exon diversity, low sequence conservation and minimal protein-coding capability. This annotation provides the first publically available baseline lncRNA database in the horse that extends across multiple tissues and individuals, providing depth and breadth, while maintaining stringent filtering criteria.

## Additional files

Additional file 1: Figure S1. Sequence conservation of equine lncRNA and protein-coding transcripts relative to mouse, cow and pig transcriptional products. Blast conservation represents the BLASTN identity multiplied by the BLASTN coverage of a given transcript. The cumulative frequency represents the percentage of lncRNA transcripts obtaining a BLASTN conservation measure equal to or less than the indicated x-axis value. (PNG 1361 kb)

Additional file 2: Table S1. The equine and corresponding human IDs of 50 lncRNA showing positional conservation to the same gene. (TXT 2 kb)

Additional file 3: Table S2. Expression table of lncRNA and protein-coding transcripts used in the expression analyses. Expression values are represented as TPM. (TXT 10830 kb)

Additional file 4: Table S3. The BED table for our final list of lncRNA. (TXT 1641 kb)

Additional file 5: Table S4. The GTF table of our final list of lncRNA. (TXT 4547 kb)

Additional file 6: Figure S2. The distribution of the candidate lncRNA across all the chromosomes, categorized by which input the lncRNA transcript originated from. The blue line represents the size of the chromosome (Mb/100,000 for scaling). (DOCX 273 kb)

## Abbreviations

ICM: Embryo inner cell mass
lncRNA: Long non-coding RNA
RNA-seq: RNA sequencing
TE: Embryo trophectoderm
TPM: Transcripts per million
