# Supplementary figures and images for: Identification of long non-coding RNA in the horse transcriptome
Source: BMC Genomics. 2017 Jul 4;18:511. doi: 10.1186/s12864-017-3884-2 (PMC5496257; doi:10.1186/s12864-017-3884-2)

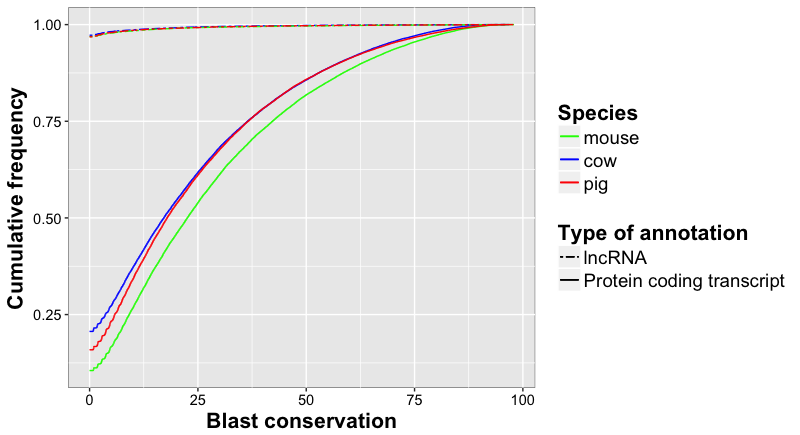

Supplement: Supplementary file 1 — Sequence conservation of equine lncRNA and protein-coding transcripts relative to mouse, cow and pig transcriptional products. Blast conservation represents the BLASTN identity multiplied by the BLASTN coverage of a given transcript. The cumulative frequency represents the percentage of lncRNA transcripts obtaining a BLASTN conservation measure equal to or less than the indicated x-axis value. (PNG 1361 kb) [file 12864_2017_3884_MOESM1_ESM.png]
