# Supplementary material for: Identification of long non-coding RNA in the horse transcriptome
Source: BMC Genomics. 2017 Jul 4;18:511. doi: 10.1186/s12864-017-3884-2 (PMC5496257; doi:10.1186/s12864-017-3884-2)
Supplement: Supplementary file 2 [file 12864_2017_3884_MOESM6_ESM.docx]

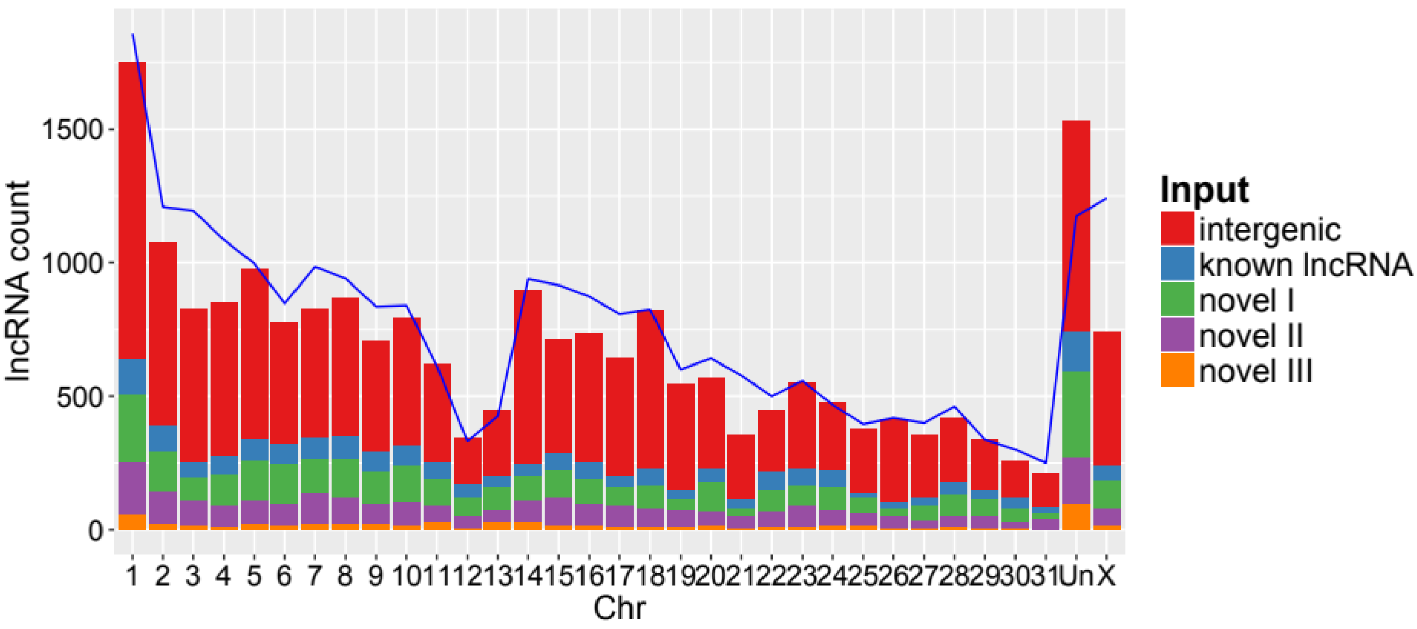

lncRNA count
1500
1000
500
0
1
2
3
4
5
6
7
8
9
10
11
12
13
14
15
16
17
18
19
20
21
22
23
24
25
26
27
28
29
30
31
Un
X
Chr
Input
intergenic
known lncRNA
novel I
novel II
novel III
